# Validation of an LC–MS/MS Method for the Quantification of the CK2 Inhibitor Silmitasertib (CX-4945) in Human Plasma

**DOI:** 10.3390/molecules27082394

**Published:** 2022-04-07

**Authors:** Rico Schwarz, Anna Richter, Elisabeth R. D. Ito, Hugo Murua Escobar, Christian Junghanß, Burkhard Hinz

**Affiliations:** 1 Institute of Pharmacology and Toxicology, Rostock University Medical Center, 18057 Rostock, Germany; rico.schwarz@med.uni-rostock.de (R.S.); e-seiler@eagle.sophia.ac.jp (E.R.D.I.); 2 Clinic for Hematology, Oncology and Palliative Care, Rostock University Medical Center, 18057 Rostock, Germany; anna.richter@med.uni-rostock.de (A.R.); hugo.murua.escobar@med.uni-rostock.de (H.M.E.); christian.junghanss@med.uni-rostock.de (C.J.)

**Keywords:** silmitasertib, CX-4945, LC–MS/MS, liquid–liquid extraction, validation, plasma

## Abstract

Silmitasertib (CX-4945) is currently being investigated in clinical trials against various types of cancer. The U.S. Food and Drug Administration (FDA) has already granted orphan drug designation to the compound for the treatment of advanced cholangiocarcinoma, medulloblastoma, and biliary tract cancer. Silmitasertib inhibits the serine/threonine protein kinase CK2, which exerts a proliferation-promoting and anti-apoptotic effect on cancer cells. In view of current and future applications, the measurement of silmitasertib levels in plasma is expected to play an important role in the evaluation of therapeutic and toxic concentrations in cancer patients. In the present work, we therefore present an LC–MS/MS method for the quantification of silmitasertib in human plasma. Using a simple liquid–liquid extraction with ethyl acetate and a mixture of n-hexane and ethyl acetate, this method can be performed in any laboratory with mass spectrometry. The validation was carried out according to the FDA guideline.

## 1. Introduction

Small-molecule kinase inhibitors have now been used clinically for two decades, starting with the approval of imatinib by the U.S. Food and Drug Administration (FDA) in 2001, and have meanwhile become a standard in modern clinical oncology (for review see [1,2]). The focus of research in this field over the last decade has included the protein kinase CK2, also known as casein kinase 2. CK2 represents a serine/threonine kinase that mediates proliferation-promoting, anti-apoptotic, and thus survival-promoting effects in cancer cells and has likewise been characterized as a regulator of angiogenesis (for review see [3,4]). Among the numerous signaling pathways regulated by CK2-mediated phosphorylation, PI3K/AKT, NF-κB, Wnt, JAK/STAT, Notch1, Hedgehog/Gli1, and p53 have been reported [5,6]. For many cancers, the overexpression of CK2 has been associated with a poor prognosis for cancer progression [7,8].

**Figure 1:**
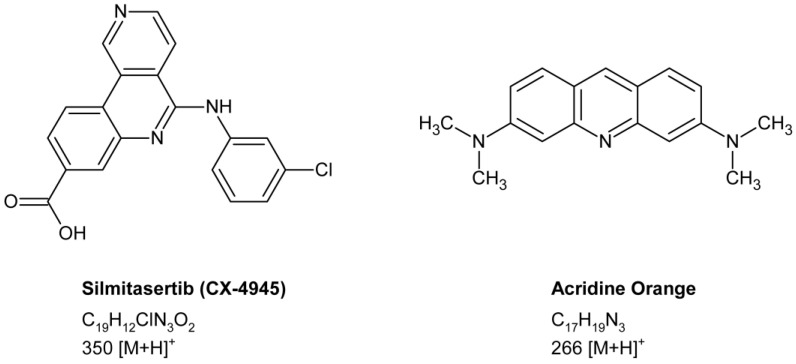
Chemical structures, molecular formulae, and precursor ions of the CK2 inhibitor silmitasertib (CX-4945) and the internal standard acridine orange.

Of the various small-molecule inhibitors of CK2, the orally bioavailable silmitasertib (CX-4945, Figure 1) has emerged as the most promising candidate. Silmitasertib inhibits CK2 with a Ki value of 0.38 nM via an ATP-competitive mechanism [9], thereby blocking downstream signaling pathways. In preclinical tests, silmitasertib showed anticarcinogenic properties in various cancer entities such as bladder [10], breast [11], gastric [12], non-small cell lung [13], pancreatic [14], and prostate cancer [15], as well as in cholangiocarcinoma [16], glioblastoma [17,18], medulloblastoma [5], and leukemia [19,20,21]. In addition, preclinical studies have shown that silmitasertib works synergistically with several classes of anticancer drugs, supporting the translation of silmitasertib combination therapies into clinical applications [22]. To accelerate the clinical development of silmitasertib, the drug received orphan drug designation from the FDA for the treatment of cholangiocarcinoma in 2016 [23], medulloblastoma in 2021 [24], and biliary tract cancer in 2022 [25]. A phase I/II study addressing the impact of silmitasertib in combination with gemcitabine and cisplatin in the treatment of patients with cholangiocarcinoma (NCT02128282) has been meanwhile completed, while clinical trials evaluating silmitasertib for the treatment of patients with basal cell carcinoma (NCT03897036), renal cancer (NCT03571438), recurrent medulloblastoma (NCT03904862), and severe COVID-19 (NCT04668209) are still recruiting (for information on studies see [26]).

In view of its already foreseeable increasing clinical use, appropriate sensitive analytical methods are essential for the measurement of silmitasertib in the context of therapeutic monitoring of cancer patients. For appropriate quantitative analyses in various test materials such as plasma or serum, liquid chromatography–tandem mass spectrometry (LC–MS/MS) is ideally suited. Of the previously published analytical methods for measuring silmitasertib in biological compartments [27,28], only one method has been validated accordingly [27]. Here, silmitasertib was determined in human plasma, cerebrospinal fluid, and brain tissue by cation-exchange solid-phase extraction followed by LC–MS/MS [27]. For easier handling by clinical laboratories, the present study focused on an alternative simple liquid–liquid extraction for the quantification of silmitasertib by LC–MS/MS in human plasma. The method has been validated according to the 2018 FDA guidance [29] and has already been successfully used to study the pharmacokinetic properties of silmitasertib in mice [30].

## 2. Results and Discussion

### 2.1. Method Development

In the currently available literature, there are two papers with methods for determining silmitasertib. The first published study investigating the pharmacokinetic properties of silmitasertib in rats examined a concentration range of 7.8–8000 ng/mL. This involved protein precipitation with acetonitrile [28]. The other method validated the determination of silmitasertib in human fluids as well as in brain tissue using solid-phase extraction. Thereby, different calibration curves in the range from 0.2 to 20,000 ng/mL were created. For brain tissue, a range from 2 to 40 ng/g was chosen [27]. The method was applied in the clinical trial NCT03904862 [31]. The method we have established and validated in a concentration range from 1.75 to 35 ng/mL, on the other hand, represents a liquid–liquid extraction that can be carried out in any laboratory at low cost. For this purpose, we optimized the liquid–liquid extraction in terms of extraction agent, time, and volume. Subsequently, the conditions for high-performance liquid chromatography (HPLC), such as suitable column and flow agent, were also optimized. Finally, the mass spectrometric parameters for the ionization and analysis of silmitasertib were adjusted.

### 2.2. Calibration Curve

**Figure 2:**
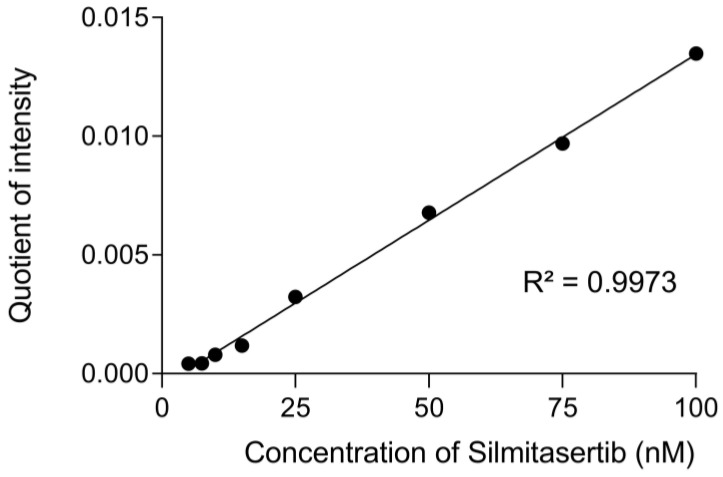
Exemplary calibration curve of silmitasertib for the concentration range 5 nM–100 nM. The values determined for the individual concentrations represent quotients of the area of the quantifiers *m*/*z* 223.1 (silmitasertib) and *m*/*z* 250.1 (internal standard).

For the calibration curve, concentrations in the range from 5 nM to 100 nM were chosen. The peak areas of the quantifiers of the internal standard and silmitasertib were correlated, and then a linear regression was performed. The coefficient of determination was at least 0.99 or better in all validation runs. An exemplary calibration curve is shown in Figure 2.

### 2.3. Selectivity and Carryover

**Figure 3:**
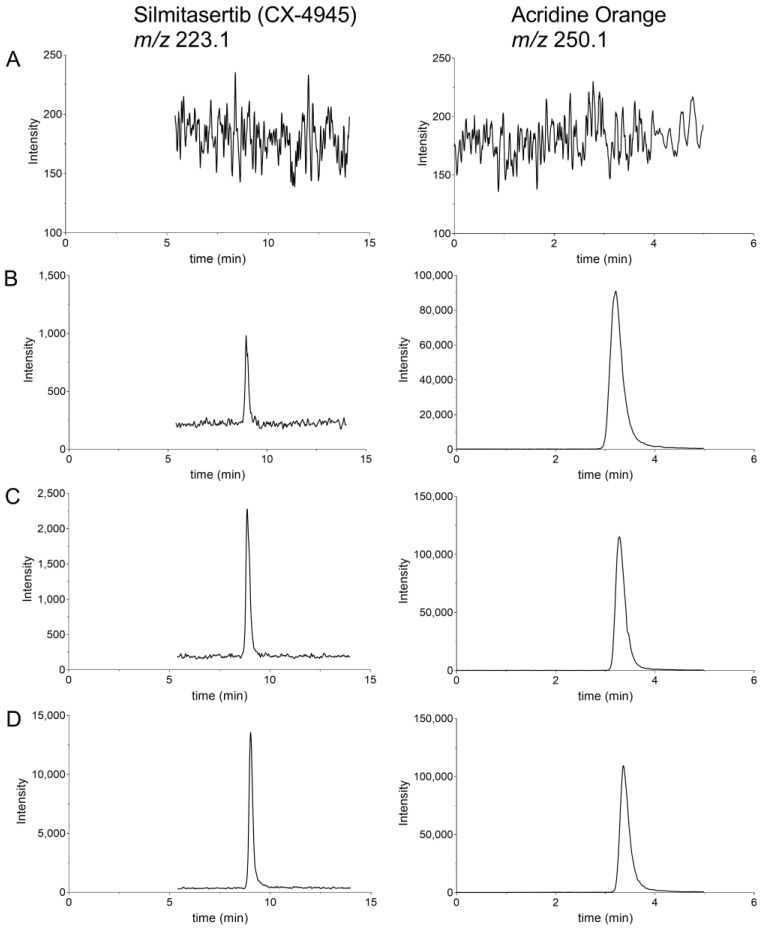
Chromatograms obtained in the LC–MS/MS analysis of (**A**) a blank sample, (**B**) a sample with 5 nM silmitasertib (LLOQ), (**C**) a 15 nM silmitasertib standard sample, and (**D**) a 100 nM silmitasertib standard sample. Shown are exemplary chromatograms of the quantifier ions for silmitasertib and the internal standard acridine orange. Not shown are the qualifier ions for silmitasertib (*m/z* 314.1) and the internal standard (*m*/*z* 234.1).

To test the selectivity of the analytical method, six different human plasma samples were examined. Thereby, no equal mass-to-charge ratio (*m*/*z*) signals were detected during the run for the retention time of silmitasertib and the internal standard acridine orange (Figure 3A). In addition, Figure 3 shows the chromatograms of the LLOQ (Figure 3B), a 15 nM standard sample (Figure 3C), and a 100 nM standard sample (Figure 3D).

To investigate sample carryover, a run with 80 µL of acetonitrile was performed between each HPLC run. No carry-over was observed.

### 2.4. Sensitivity, Accuracy, and Precision

The LLOQ determined for silmitasertib was 5 nM, which corresponds to 4 pmol or 1.4 ng of absolute substance at an injection volume of 80 µL. The signal-to-noise ratio (S/N) of the LLOQ was at least 5 in each validation run.

The inter- and intra-day precisions of the LLOQ and the concentrations of 15 nM (low level), 50 nM (medium level), and 100 nM (high level) are shown in Table 1. The FDA requirements that the relative deviations in each validation run should not differ by more than ±15% or ±20% for the LLOQ [29] were always met. The coefficient of variation (CV) as a measure of the precision of the measured concentrations ranged from 7.15% for 15 nM (L) to 9.08% for 50 nM (M) for the inter-day studies. However, the CV in each validation run (intra-day) was within the FDA specifications of ±15% or ±20% for the LLOQ [29].

### 2.5. Matrix Effect and Recovery

Taking into account that plasma components can negatively influence the electrospray ionization of other components in the source (for an overview see [32]), several human plasma samples of different quality were investigated for the determination of the recovery and the matrix effect. For determination of the matrix effect, the matrix was extracted and then spiked with silmitasertib (*n* = 5). These samples were compared with non-extracted working solutions, and the absolute peak areas of the samples were related. An ion suppression was the result (Table 2). The matrix effect ranged from 18.36 ± 10.09% (100 nM) to 26.57 ± 12.44% (50 nM) and to 33.95 ± 7.49% (15 nM) with mean ± SD of *n* = 5 indicated here. A higher matrix effect was observed especially at the lower concentration of 15 nM, but without any effect on the calibration functions.

The recovery of silmitasertib was investigated by spiking plasma with 15 nM (low concentration), 50 nM (medium concentration), and 100 nM silmitasertib (high concentration) and then processing (*n* = 4). The areas of the samples spiked with silmitasertib and subsequently extracted were compared with the extracted matrix samples spiked with silmitasertib after the extraction step (Table 2). On average, the recovery from the 15 nM spiked sample was 56.89 ± 8.44% with a precision (CV) of 14.84%. Similar precisions were determined for the 50 nM spiked sample, with 15.47% CV (average recovery of 47.79 ± 7.39%), and for the 100 nM spiked sample, with 17.89% CV (average recovery of 57.24 ± 10.24%). The recovery was approximately the same over the concentration range investigated. However, it varied by about 15% regardless of the concentration. Therefore, silmitasertib was recovered at about 50% from samples at all three concentration levels investigated. The mean recovery of the internal standard acridine orange was 59.02 ± 3.28%, with a CV of 5.56% (*n* = 5). In fact, the recovery values for silmitasertib as well as for the internal standard were relatively low. However, an inefficient extraction could be excluded in preliminary tests by additional extraction steps. Therefore, the influence of matrix components on the extraction as well as the ionization of silmitasertib and the internal standard could be possible reasons for the low recovery. Irrespective of the comparatively low recovery, this parameter proved to be reproducible, meeting the requirements of the FDA for method validation.

### 2.6. Stability

**Figure 4:**
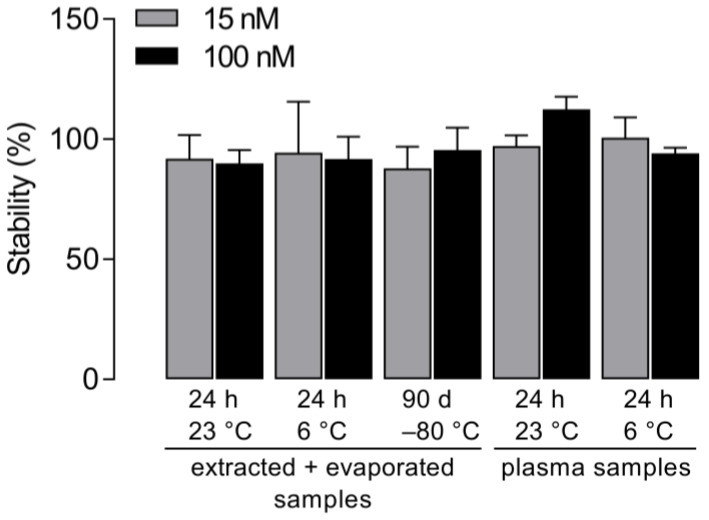
Stability of silmitasertib as a function of time and temperature of storage, relative to freshly extracted controls on the day of measurement (set to 100%). The ratio of the *m/z* values of the quantifiers *m/z* 223.1 (silmitasertib) and *m/z* 250.1 (internal standard) was determined. Shown are mean values ± SD of *n* = 3 independent LC–MS/MS runs.

In a recently published study, the stability of silmitasertib was investigated in various human fluids and brain homogenates as well as in stock solutions. Thereby, a high stability of silmitasertib could be demonstrated under different conditions and when using different matrices [27]. In the present study, stability studies were carried out in human plasma. In addition, the stability of silmitasertib in extracted and evaporated samples was investigated, with particular focus on these types of samples in the long-term stability tests conducted. Stability was investigated using silmitasertib concentrations of 15 nM (low concentration) and 100 nM (high concentration). The samples were stored for 24 h either at 23 °C room temperature or at 6 °C in a refrigerator. In addition, long-term stability was investigated over a period of 90 days at –80 °C (Figure 4). For comparison, fresh samples were extracted on the day of measurement before the stored samples were analyzed. Silmitasertib was considered stable if its nominal concentration did not vary by more than ±15%.

After extraction and evaporation, silmitasertib was stable over 90 days. During storage at 23 °C and 6 °C, small losses of about 15% were observed in the evaporated and extracted samples (Figure 4). Silmitasertib was stable when stored in human serum at both 23 °C and 6 °C.

### 2.7. Quantification of Silmitasertib in Artificial Mouse Plasma

Finally, experiments were carried out with artificial mouse plasma to apply the method. For this purpose, 990 µL of human plasma was mixed with 10 µL of artificial mouse plasma, which contained 5 µM silmitasertib, to obtain a final volume of 1 mL. After extraction of the plasma sample, thus finally spiked with 50 nM silmitasertib, the analyte was detected and quantified by LC–MS/MS. The experiments revealed a silmitasertib concentration of 50.61 nM ± 3.53 nM (mean ± SD, *n* = 6) with a CV of 6.97%. As mentioned above, the method was also used in a previously published study measuring silmitasertib serum levels in mice treated intraperitoneally with the CK2 inhibitor [30].

## 3. Materials and Methods

### 3.1. Chemical Reagents

Silmitasertib (CX-4945) was purchased from Merck Millipore (Darmstadt, Germany) with a purity of ≥98% and was dissolved in DMSO as a 10 mM stock solution. Acridine orange, LiChrosolv^®^ water, and lyophilized mouse plasma were likewise obtained from Merck Millipore (Darmstadt, Germany), formic acid from Honeywell Fluka (Seelze, Germany), and acetonitrile ultragradient HPLC-grade from J. T. Baker (Gliwice, Poland).

### 3.2. Standard Preparation

Calibration curves from 5 nM to 100 nM (5, 7.5, 10, 15, 25, 50, 75, and 100 nM) were prepared for silmitasertib by adding 10 μL of an appropriate working solution to 1 mL of human plasma followed by extraction and mass spectrometric analysis. The working solutions were prepared by a serial dilution series from the 10 mM stock solution (dissolved in DMSO) in acetonitrile. Then, 10 µL of a 5 µM working solution (dissolved in water) of the internal standard acridine orange was added to these solutions with a corresponding final concentration of acridine orange of 50 nM. The standard solutions were extracted three times with 1 mL of extraction solution. The first step was carried out with pure ethyl acetate as the extraction agent, the other two steps with a mixture of ethyl acetate/n-hexane (2:1, *v*/*v*). The organic phase was evaporated to dryness using a vacuum concentrator (SpeedVac SPD130DLX, Thermo Fisher Scientific, Asheville, NC, USA). The residues were reconstituted for LC–MS/MS measurements in 100 μL water/acetonitrile (65:35, *v*/*v*) mixed with 0.2% formic acid.

### 3.3. LC–MS/MS Analysis

From the extracted and reconstituted samples, 80 µL was separated using a Shimadzu LC-20AD HPLC with a Multospher 120 C18 AQ column 125 × 2 mm, 5 µm particle size (CS-Chromatographie Service GmbH, Langerwehe, Germany) coupled with a guard column 20 mm × 3 mm, 5 µm particle size. Silmitasertib and the internal standard were analyzed with water containing 0.2% formic acid (mobile phase A) and acetonitrile containing 0.2% formic acid (mobile phase B) at a flow rate of 0.3 mL/min. The following solvent compositions were chosen: the starting gradient was maintained at 65% of mobile phase A for 2 min, then mobile phase B was linearly increased to 100% within 20 min, after which the starting conditions were immediately adjusted and maintained for a further 13 min to restore equilibrium. The eluate was transferred via HPLC to a Micromass Quatro Micro^TM^ API mass spectrometer (Waters, Milford, MA, USA), and the eluted substances were ionized by electrospray ionization in positive mode. Multiple reaction monitoring (MRM) was used to quantify the substances. The MS/MS transitions for the precursor ion at *m/z* 266.11 for the internal standard acridine orange were *m/z* 234.1 at 44 V collision energy at a cone voltage of 24 V and *m/z* 250.1 at 24 V collision energy. For the precursor ion of silmitasertib at *m/z* 350.04 at a cone voltage of 40 V, two MS/MS transitions were used. The first was *m/z* 223.1 at 12 V collision energy, and the second was *m/z* 314.1 at 24 V collision energy. The quantifier for silmitasertib was *m/z* 223.1 and for the internal standard acridine orange, it was *m/z* 250.1. The other MS/MS transitions were used as qualifiers. Further mass spectrometer values and source parameters were as follows: capillary voltage 3.5 kV, source temperature 120 °C, evaporation temperature 350 °C, evaporation gas flow rate 550 L h^−1^, and cone gas flow rate 50 L h^−1^. Dwell and delay times were 0.05 and 0.1 s, respectively. Argon was used as a collision gas for collision-induced dissociation (CID). The instrument parameters for the internal standard and for silmitasertib were adjusted by injection of a 10 µM standard solution with a flow rate of 10 µL min^−1^ with a syringe pump. Data acquisition was done with the software MassLynx Version 4.1 (Waters, Milford, MA, USA) and LabSolutions Version 5.57 (Shimadzu, Duisburg, Germany).

### 3.4. Validation

The method validation was performed in accordance with the FDA guidelines for bioanalytical methods of 2018 [29]. In this context, the parameters calibration curve, selectivity, sensitivity, accuracy, precision, recovery, matrix effect, and stability were determined for silmitasertib in the present study. Human plasma was chosen as the matrix. The method was validated using the ratios of the areas under the curve of the analyte quantifier and the internal standard acridine orange. The calibration values were compared with the method of least squares.

A range of 5 nM to 100 nM (5 nM, 7.5 nM, 10 nM, 15 nM, 25 nM, 50 nM, 75 nM, 100 nM) was chosen for the concentrations of the calibration curves for silmitasertib. In each validation run, 75% of these concentrations and at least 6 non-zero calibrators had to be used. Silmitasertib was used in concentrations of 15 nM (low concentration), 50 nM (medium concentration), and 100 nM (high concentration) for the determination of each parameter. The concentration of the internal standard acridine orange was set constantly at 50 nM.

To test the selectivity of the method, six different human plasma samples were analyzed.

The sensitivity was determined by evaluating the lower limit of quantification (LLOQ), whose S/N had to be at least 5 [29]. The S/N ratio was determined with the software MassLynx Version 4.1 without prior smoothing.

For accuracy, expressed as relative error (RE; [(measured concentration − nominal concentration)/nominal concentration] × 100%), a deviation of ±20% for the LLOQ and of ±15% of their nominal concentrations for the other concentrations used was allowed [29]. As a measure of precision, the CV (standard deviation/mean of the measured concentration × 100%) was set at ±20% for the LLOQ and at ±15% for the other concentrations used. Both parameters were determined as inter- and intra-day using five samples on three different days.

The matrix effect was determined by analyzing five samples with low (15 nM), medium (50 nM), and high (100 nM) concentration. For the determination, extracted matrix samples post-spiked with silmitasertib were compared with non-extracted standard solutions according to the following formula: [(1 − (post-spiked signal/stock solution signal)] × 100%.

To determine the recovery of silmitasertib, four independent samples from different human plasma samples were analyzed. The recovery was investigated with low (15 nM), medium (50 nM), and high (100 nM) concentrations. Extracted matrix samples spiked with the three concentrations prior to measurement were analyzed as reference values. Recovery was determined by comparing the peak areas of the extracted samples with those of the corresponding extracts of blank samples spiked with the analyte after extraction [(sample signal/post spiking signal) × 100%]. The internal standard was added before the analysis.

Stability was investigated using various parameters, with samples after extraction and evaporation and with samples dissolved in the matrix. Short-term stability was assessed by storing the samples for 24 h at 23 °C as well as at 6 °C. Long-term stability was studied with extracted samples stored at −80 °C for 90 days. The peak area ratios of analyte and internal standard of the quantifiers of the stability experiments were compared with those of the freshly extracted samples evaporated on the day of measurement. The internal standard was always added to all samples before the analysis. To avoid possible concentration fluctuations due to the possible degradation of silmitasertib, stock solutions were prepared monthly and stored at −20 °C.

### 3.5. Preparation of Artificial Mouse Plasma

To establish the method, silmitasertib was added to artificial mouse plasma to a final concentration of 5 µM. Then, 10 µL of the silmitasertib-containing artificial mouse plasma was added to human plasma with a resulting final volume of 1 mL, yielding a final silmitasertib concentration of 50 nM. To generate the calibration function, artificial mouse plasma was also diluted by using the same human plasma. In this way, the matrix effect was kept as low as possible. The internal standard was added to the mixture. The samples were then extracted and analyzed as described above.

## 4. Conclusions

Silmitasertib (CX-4945) is a promising CK2 inhibitor that shows antiproliferative and proapoptotic effects in preclinical models testing various cancer entities and has since received FDA Orphan Drug designations for the treatment of cholangiocarcinoma, medulloblastoma, and biliary tract cancer. Due to the expected broad application of silmitasertib in the future, concentration determination in biological matrices is crucial for the evaluation of this antitumor therapy. In the present study, we presented a validated LC–MS/MS method for the quantification of silmitasertib in human plasma based on a simple-to-perform triple liquid–liquid extraction. This method can thus be used in any mass spectrometry laboratory. The calibration function was chosen in a low concentration range from 5 nM up to 100 nM, so that only small plasma sample volumes of the treated subjects are required. The recovery was determined to be about 50% in all plasma samples tested. We were able to successfully apply the method to the analysis of silmitasertib in artificial mouse plasma previously spiked with the substance or in mouse sera from animals treated intraperitoneally with the CK2 inhibitor [30].

## Figures and Tables

**Table 1:** Inter-day and intra-day accuracy, expressed as relative error (RE) ± standard deviation (SD) and precision, determined as coefficient of variation (CV), of five different samples, each from three days of silmitasertib at LLOQ level (5 nM), low (15 nM), medium (50 nM), and high (100 nM) concentrations. The measured concentrations are given as mean values ± SD; *n* = 5 for intra-day, *n* = 15 for inter-day.

Used Concentration (nM)	Intra-Day (Day 1)			Intra-Day (Day 2)			Intra-Day (Day 3)			Inter-Day	
	Measured Concentration (nM)	RE (%)	CV (%)	Measured Concentration (nM)	RE (%)	CV (%)	Measured Concentration (nM)	RE (%)	CV (%)	RE (%)	CV (%)
5	5.20 ± 0.34	4.03 ± 6.77	6.51	5.16 ± 0.42	3.28 ± 8.44	8.17	5.16 ± 0.59	3.27 ± 11.82	11.45	3.53 ± 8.57	8.28
15	15.84 ± 0.96	5.60 ± 6.37	6.03	15.46 ± 1.18	3.07 ± 7.88	7.64	15.38 ± 1.37	2.54 ± 9.12	8.90	3.74 ± 7.42	7.15
50	51.50 ± 5.39	3.01 ± 10.78	10.47	53.18 ± 3.01	6.36 ± 6.01	5.65	46.31 ± 1.71	−7.37 ± 3.42	3.69	0.67 ± 9.14	9.08
100	106.47 ± 3.24	6.47 ± 3.24	3.04	101.00 ± 8.22	1.00 ± 8.22	8.24	97.97 ± 7.98	−2.03 ± 7.98	8.14	1.81 ± 7.37	7.23

**Table 2:** Matrix effect and recovery of silmitasertib and the internal standard acridine orange. Values are mean percentages ± standard deviation (SD) of *n =* 5 (except for the recovery of silmitasertib, *n* = 4).

Silmitasertib			Acridine Orange	
Concentration (nM)	Recovery (%)	Matrix Effect (%)	Concentration (nM)	Recovery (%)
15	56.89 ± 8.44	33.95 ± 7.49	50	59.02 ± 3.28
50	47.79 ± 7.39	26.57 ± 12.44		
100	57.24 ± 10.24	18.36 ± 10.09		

## Data Availability

The data presented in this study are available on request from the first author.

## Sample Availability

Not available.
